# Human Activity Recognition with an HMM-Based Generative Model

**DOI:** 10.3390/s23031390

**Published:** 2023-01-26

**Authors:** Narges Manouchehri, Nizar Bouguila

**Affiliations:** 1 Algorithmic Dynamics Lab, Unit of Computational Medicine, Karolinska Institute, 171 77 Stockholm, Sweden; 2 Concordia Institute for Information Systems Engineering, Concordia University, Montreal, QC H3G1T7, Canada

**Keywords:** human activity recognition, scaled Dirichlet distribution, hidden Markov models, proportional data, medical applications

## Abstract

Human activity recognition (HAR) has become an interesting topic in healthcare. This application is important in various domains, such as health monitoring, supporting elders, and disease diagnosis. Considering the increasing improvements in smart devices, large amounts of data are generated in our daily lives. In this work, we propose unsupervised, scaled, Dirichlet-based hidden Markov models to analyze human activities. Our motivation is that human activities have sequential patterns and hidden Markov models (HMMs) are some of the strongest statistical models used for modeling data with continuous flow. In this paper, we assume that emission probabilities in HMM follow a bounded–scaled Dirichlet distribution, which is a proper choice in modeling proportional data. To learn our model, we applied the variational inference approach. We used a publicly available dataset to evaluate the performance of our proposed model.

## 1. Introduction

Human activity recognition (HAR) has received significant attention in recent years. This topic is helping scientists to understand behaviors, anticipate intentions, predict probable actions, monitor healthcare, and assist in rehabilitation [1,2,3,4,5,6,7]. To collect data for discovering the patterns of human activities, various devices (e.g., advanced electronic devices and smart technologies) have been developed. This resulted in large amounts of data. HAR data have been conventionally collected by cameras and sensors [8,9,10,11]. As vision-based data could be collected easier compared to sensor-based data, they have been used previously. However, they have some critical challenges in instance-privacy issues. In contrast, cheap sensors do not have such disadvantages and became more practical methods used in collecting information. These tools have various types of sensors, such as wearable, ambient, and object-tagged sensors [12,13,14,15,16,17,18]. After collecting data, we need to choose an adequate model to capture the hidden patterns of human activities. In HAR, we face some challenges, such as difficulties in associating activities with various users, various patterns and styles for a single activity, similarities in activities, unpredictable and accidental events, noise, and difficulties in labeling. To overcome these challenges, several machine learning methods have been introduced [19,20,21,22,23,24,25]. Some platforms, such as deep learning, provide better results [26,27,28,29,30,31,32,33]. However, these approaches are data-hungry and they need considerable amounts of labeled data. Data annotation is another difficulty as it is time-consuming and expensive. Another problem is that deep learning platforms are not absolutely explainable in human terms [34,35,36,37,38,39,40,41,42,43,44,45,46,47]. HMMs are less computationally expensive compared to deep learning and could be easily adapted for real-time scenarios. Considering the discussed challenges, we focused on generative models. Hidden Markov models are powerful when dealing with temporal data. In the case of continuation in receiving data points and the interest in finding hidden states by modeling observable sequential data, HMM is a capable model. HMM has been used in various medical applications [48,49,50,51,52,53,54,55,56,57,58,59,60,61,62]. Moreover, it has been applied in human activity recognition [63,64,65,66,67,68,69,70,71,72,73,74,75,76,77,78,79,80,81]. In HMM, it is commonly assumed that emission probabilities are raised from Gaussian mixture models (GMM) [82,83,84,85,86,87]. However, the assumption of Gaussianity could not be valid for all cases. As a consequence, other alternatives and distributions have been applied [88]. In this work, we assume that emission probability distributions follow scaled Dirichlet mixture models, which improve the structure of HMM. In fact, scaled Dirichlet distribution is a proper choice for modeling proportional data [89]. We call our proposed model scaled Dirichlet HMM (SD-HMM). We apply variational inference (VI) as an elegant learning method for estimating model parameters.

VI is an approximation technique, more accurate compared to ML and faster than fully Bayesian inference [90,91,92,93,94,95,96,97,98]. Moreover, compared to a deterministic method, such as maximum likelihood, it does not suffer from convergence to a local maximum and over-fitting. Moreover, it is not as computationally complex as fully Bayesian. Finally, we evaluate our proposed model, SD-HMM, on publicly available datasets of human activity recognition. To evaluate our model, we compared it with the hidden Markov model with GMM emission probabilities as a widely used alternative.

In this work, we propose an improved version of the hidden Markov model, such that emission probabilities are raised from scaled Dirichlet mixture models. Then, we applied an elegant learning method, variational inference, to estimate parameters. we evaluated the performance of our proposed model and compared it with similar alternatives in human activity recognition.

The paper is organized as follows: In Section 2, we discuss HMM. In Section 3, we estimate model parameters with variational inferences. In Section 4, we present the results of evaluating our proposed model in human activity recognition. We conclude in Section 6.

## 2. Hidden Markov Model

In this section, we explain the first Markov chain. Let us consider a sequence of states or events. In the first-order Markov Model, it is assumed that the future state depends only on the current state. Thus, an event at a particular point in time *t* depends on the previous event at time t−1. HMM is expressed by the following parameters:

- Transition probability, which is the probability of altering the state at time *t* to the same state or another state at the time step t+1. The sum of all transition probabilities at the current state is equal to 1.
- Emission probability, which indicates the probability of an observation generated from a particular state.
- Initial probability π, in which HMM starts at time step of 0. The sum of all probabilities is equal to 1.

We express HMM with parameters θ={B,C,φ,π} by the following notations:

- A sequence of observations: $X=\{X_{1},\cdots ,X_{T}\}$ generated by hidden states $H=\{h_{1},\ldots ,h_{T}\}$ $h_{j}\in \left[1,K\right]$. *K* indicates the number of states.
- Transition matrix: $B=\{b_{jj^{\prime }}=P\left(h_{t}=j^{\prime }|h_{t-1}=j\right)\}$.
- Emission matrix: C= $\left\{C_{ij}=P\left(m_{t}=j|h_{t}=i\right)\right\}$ for j∈[1,M]. *M* indicates the number of mixture components associated with the state *j*.
- $\pi_{j}$: Initial probability to start the sequence from state *j*.

φ includes two parameters of the SD mixture model. To modify conventional HMM, we first explain the SD distribution. Let us consider a *D*-dimensional observation $\vec{X}=\left(x_{1},\cdots ,x_{d}\right)$, which is drawn from the SD distribution with two parameters, $\vec{\alpha }$ and $\vec{\beta }$.

(1) $$\begin{array}{c} \mathrm{SD}\left({\vec{X}}_{n}|\varphi \right)=\frac{\Gamma (\sum_{d=1}^{D}\alpha_{d})}{\prod_{d=1}^{D}\Gamma \left(\alpha_{d}\right)}\frac{\prod_{d=1}^{D}\beta_{d}^{\alpha_{d}}x_{d}^{\alpha_{d}-1}}{{\left(\sum_{d=1}^{D}\beta_{d}x_{d}\right)}^{\sum_{d=1}^{D}\alpha_{d}}} \end{array}$$

Γ is the Gamma function. $\sum_{d=1}^{D}\alpha_{d}=\sum_{d=1}^{D}\alpha_{d}$ and $\varphi =(\vec{\alpha },\vec{\beta })$ where $\vec{\alpha }=\left(\alpha_{1},\ldots ,\alpha_{D}\right)$ and $\vec{\beta }=\left(\beta_{1},\ldots ,\beta_{D}\right)$ are respective shape and scale parameters. These two parameters provide more flexibility in modeling various shapes of data. Moreover, we are assuming that $\vec{X}$ is a proportional vector. To express the modification of HMMs, we represent the estimates of states and mixture components by:

(2) $$\begin{array}{c} \gamma_{h_{t},m_{t}}^{t}≜p\left(h_{t},m_{t}|x_{0},\ldots ,x_{T}\right) \end{array}$$

and the local states sequence given the whole observation set by:

(3) $$\begin{array}{c} \xi_{ht,ht+1}^{t}≜p\left(h_{t},h_{t+1}|x_{0},\ldots ,x_{T}\right) \end{array}$$

$\gamma_{h_{t},m_{t}}^{t}$ and $\xi_{h_{t},h_{r}+1}^{t}$ for all t∈[1,T] are responsibilities and computed by similar forward–backward procedures for HMM with Gaussian mixtures.

## 3. Parameter Estimation with Variational Inference

In this section, we discuss a powerful learning approach, variational inference, which inherits the advantages of deterministic and Bayesian inferences. This technique has more precise results compared to deterministic methods while being faster than fully Bayesian inference. The idea is to minimize the distance between the true posterior and an approximating distribution using the Kullback–Leibler (KL) divergence [90]. Moreover, With this approximation scheme, we can simultaneously estimate the model’s parameters and the optimal number of components. Here, we will explain the steps of variational learning to update all parameters of HMM for the transition distribution, mixing matrix, and shape parameters of emission distributions. As the first step, we need to define a prior distribution for each parameter. Given the model parameters, the likelihood of sequential observations $\vec{X}$ is defined as follows, where *S* and *L* are the respective sets of hidden states and the set of mixture components:

(4) $$\begin{array}{c} p\left(\vec{X}|B,C,\pi ,\varphi \right)=\sum_{S}\sum_{L}\pi_{s_{1}}\left[\prod_{t=2}^{T}b_{s_{t-1},s_{t}}\right]\left[\prod_{t=1}^{T}c_{s_{t},m_{t}}p\left(x_{t}|\varphi_{s_{t},m_{t}}\right)\right] \end{array}$$

(5) $$\begin{array}{c} p\left(\vec{X}\right)=\int d\pi dbdCd\varphi \sum_{S,L}p\left(B,C,\pi ,\varphi \right)p\left(\vec{X},S,L|B,C,\pi ,\varphi \right) \end{array}$$

Considering that this quantity is not computable, we introduce a lower bound by using an approximating distribution q(B,C,π,φ,S,L) of the true posterior $p(B,C,\pi ,\varphi ,S,L|\vec{X})$. So,

(6) $$\begin{array}{cc} & \ln\left(p\left(\vec{X}\right)\right)=\ln\left\{\int dBdCd\pi d\varphi \sum_{S,L}p\left(B,C,\pi ,\varphi \right)p\left(\vec{X},S,L|B,C,\pi ,\varphi \right)\right\}\\ & \geq \int d\pi dBdCd\alpha \sum_{S,L}q\left(B,C,\pi ,\varphi ,S,L\right)\ln\left\{\frac{p\left(B,C,\pi ,\varphi \right)p\left(\vec{X},S,L|B,C,\pi ,\varphi \right)}{q(B,C,\pi ,\varphi ,S,L)}\right\} \end{array}$$

Further to Jensen’s inequality and considering that KL(q∣∣p)≥0, KL(q∣∣p)=0 if *q* is equal to the true posterior. L(q) is a lower bound to $\ln p(\vec{X})$:

(7) $$\begin{array}{c} \ln\left(p\left(\vec{X}\right)\right)=L\left(q\right)-\mathrm{KL}\left(q\left(B,C,\pi ,\varphi ,S,L\right)∥p\left(B,C,\pi ,\varphi ,S,L|\vec{X}\right)\right) \end{array}$$

Now, with the help of the mean field theory, we define a restricted family of distributions to compute the true posterior distribution as it is not tractable:

(8) $$\begin{array}{c} q(B,C,\pi ,\alpha ,\beta ,S,L)=q(B)q(C)q(\pi )q(\alpha )q(\beta )q(S,L) \end{array}$$

We define the lower bound as follows:

(9) $$\begin{array}{l} \ln\left(p\left(\vec{X}\right)\right)\geq \sum_{S,L}\int dBdCd\pi d\alpha d\beta q\left(\pi \right)q\left(B\right)q\left(C\right)q\left(\alpha \right)q\left(\beta \right)q\left(S,L\right)\{\ln\left(p\left(\pi \right)\right)+\ln\left(p\left(B\right)\right)\\ +\ln\left(p\left(C\right)\right)+\ln\left(p\left(\alpha \right)\right)+\ln\left(p\left(\beta \right)\right)+\ln\left(\pi_{s_{1}}\right)+\sum_{t=2}^{T}\ln\left(a_{s_{t-1},s_{t}}\right)+\sum_{t=1}^{T}\ln\left(c_{s_{t},m_{t}}\right)\\ +\sum_{t=1}^{T}\ln\left(p\left(x_{t}|\alpha_{s_{t},m_{t}}\right)\right)-\ln\left(q\left(S,L\right)\right)-\ln\left(q\left(\pi \right)\right)-\ln\left(q\left(B\right)\right)-\ln\left(q\left(C\right)\right)\\ -\ln(q(\alpha ))\}=F(q(\pi ))+F(q(C))+F(q(B))+F(q(\alpha ))+F(q(\beta ))+F(q(S,L)) \end{array}$$

To define the priors for HMM parameters, we choose the Dirichlet distribution for B,C, and π, considering that all of the coefficients of these parameters are strictly positive, less than one, and their coefficients sum up to one.

(10) $$\begin{array}{c} p\left(\pi \right)=D\left(\pi |\phi^{\pi }\right)=D\left(\pi_{1},\ldots ,\pi_{K}|\phi_{1}^{\pi },\ldots ,\phi_{K}^{\pi }\right) \end{array}$$

(11) $$\begin{array}{c} p\left(B\right)=\prod_{i=1}^{K}D\left(b_{i_{1}},\ldots ,b_{i_{K}}|\phi_{i_{1}}^{B},\ldots ,\phi_{i_{K}}^{B}\right) \end{array}$$

(12) $$\begin{array}{c} p\left(C\right)=\prod_{i=1}^{M}D\left(c_{i_{1}},\ldots ,c_{i_{M}}|\phi_{i_{1}}^{C},\ldots ,\phi_{i_{M}}^{C}\right) \end{array}$$

For α and β, we choose Gamma and Dirichlet distributions, respectively:

(13) $$\begin{array}{c} p\left(\alpha_{jd}\right)=G\left(\alpha_{jd}|u_{jd},v_{jd}\right)=\frac{v_{jd}^{u_{jd}}}{\Gamma \left(u_{jd}\right)}\alpha_{jd}^{u_{jd}-1}e^{-v_{jd}\alpha_{jd}} \end{array}$$

(14) $$\begin{array}{c} p\left({\vec{\beta }}_{j}\right)=D\left(\vec{\beta }j|{\vec{h}}_{j}\right)=\frac{\Gamma \left(\sum_{d=1}^{D}h_{jd}\right)}{\prod_{d=1}^{D}\Gamma \left(h_{jd}\right)}\prod_{d=1}^{D}\beta_{jd}^{h_{jd}-1} \end{array}$$

${\vec{h}}_{j}=\left(h_{j1},\ldots ,h_{jD}\right)$. Gamma and Dirichlet distributions are defined as G(.) and D(.), respectively. Moreover, $\left\{u_{jd}\right\},\left\{v_{jd}\right\}$, and $\left\{h_{jd}\right\}$ indicate positive hyperparameters.

Now, we explain the optimization of q(B),q(C), and q(π) as follows:

(15) $$\begin{array}{cc} & F\left(q\left(B\right)\right)=\int dBq\left(B\right)\ln\left[\frac{\prod_{i=1}^{K}\prod_{j=1}^{K}b_{ij}^{w_{ij}^{B}-1}}{q(B)}\right] \end{array}$$

(16) $$\begin{array}{c} w_{ij}^{B}=\sum_{t=2}^{T}\gamma_{ijt}^{B}+\phi_{ij}^{B} \end{array}$$

(17) $$\begin{array}{c} \gamma_{ijt}^{B}≜q\left(s_{t-1}=i,s_{t}=j\right) \end{array}$$

(18) $$\begin{array}{c} q\left(B\right)=\prod_{i=1}^{K}D\left(b_{i1},\ldots ,b_{iK}|w_{i1}^{B},\ldots ,w_{iK}^{B}\right) \end{array}$$

(19) $$\begin{array}{cc} & q\left(\pi \right)=D\left(\pi_{1},\ldots ,pi_{K}|w_{1}^{\pi },\ldots ,w_{K}^{\pi }\right) \end{array}$$

(20) $$\begin{array}{c} w_{i}^{\pi }=\gamma_{i}^{\pi }+\phi_{i}^{\pi } \end{array}$$

(21) $$\begin{array}{c} \gamma_{i}^{\pi }≜q\left(s_{1}=i\right) \end{array}$$

(22) $$\begin{array}{cc} & q\left(C\right)=\prod_{i=1}^{K}D\left(c_{i1},\ldots ,c_{iM}|w_{i1}^{C},\ldots ,w_{iM}^{C}\right) \end{array}$$

(23) $$\begin{array}{c} w_{ij}^{C}=\sum_{t=1}^{T}\gamma_{ijt}^{C}+\phi_{ij}^{C} \end{array}$$

(24) $$\begin{array}{c} \gamma_{ijt}^{C}≜q\left(s_{t}=i,m_{t}=j\right) \end{array}$$

For q(α) and q(β):

(25) $$\begin{array}{c} Q\left(\vec{\alpha }\right)=\prod_{j=1}^{M}\prod_{d=1}^{D}G\left(\alpha_{jd}|u_{jd}^{*},v_{jd}^{*}\right) \end{array}$$

(26) $$\begin{array}{c} Q\left(\vec{\beta }\right)=\prod_{j=1}^{M}\prod_{d=1}^{D}D\left(\beta_{jd}|h_{jd}^{*}\right) \end{array}$$

(27) $$\begin{array}{c} u_{jd}^{*}=u_{jd}+\eta_{jd},\;v_{jd}^{*}=v_{jd}-\vartheta_{jd} \end{array}$$

(28) $$\begin{array}{c} \eta_{jd}=\sum_{i=1}^{N}\langle Z_{pij}\rangle {\bar{\alpha }}_{jd}\left[\psi \left(\sum_{d=1}^{D}{\bar{\alpha }}_{jd}\right)-\psi \left({\bar{\alpha }}_{jd}\right)\right.\left.+\sum_{d\neq s}^{D}\psi^{\prime }\left(\sum_{d=1}^{D}{\bar{\alpha }}_{jd}\right)\times {\bar{\alpha }}_{js}\left(\langle \ln\alpha_{js}\rangle -\ln{\bar{\alpha }}_{js}\right)\right] \end{array}$$

(29) $$\begin{array}{c} \vartheta_{jd}=\sum_{i=1}^{N}\langle Z_{pij}\rangle \left[\ln{\bar{\beta }}_{jd}+\ln X_{id}-\ln\left(\sum_{d=1}^{D}{\bar{\beta }}_{jd}X_{id}\right)\right] \end{array}$$

(30) $$\begin{array}{c} h_{jd}^{*}=h_{jd}+\tau_{jd} \end{array}$$

(31) $$\begin{array}{c} \tau_{jd}=\sum_{i=1}^{N}\langle Z_{pij}\rangle \left[{\bar{\alpha }}_{jd}-{\bar{\alpha }}_{jd}{\bar{\beta }}_{jd}\frac{X_{id}}{\sum_{d=1}^{D}{\bar{\beta }}_{jd}X_{id}}\right] \end{array}$$

If $X_{pt}$ is assigned to state *i* and mixture component *j*, $Z_{pij}=1$, otherwise, zero. To compute responsibilities, we apply a typical forward–backward procedure [99] where $\langle Z_{pij}\rangle =\sum_{t=1}^{T}\gamma_{pijt}^{C}=p\left(s=i,m=j|X\right)$.

In the expectation step, we keep fixed the parameters estimated in the previous step, and the updated values are computed as follows:

(32) $$\begin{array}{cc} \pi_{i}^{⋆} & ≜\exp\left[{\langle \ln\left(\pi_{i}\right)\rangle }_{q(\pi )}\right]=\exp\left[\Psi \left(w_{i}^{\pi }\right)-\Psi \left(\sum_{i}w_{i}^{\pi }\right)\right] \end{array}$$

(33) $$\begin{array}{cc} b_{jj^{\prime }}^{⋆} & ≜\exp\left[{\langle \ln\left(b_{jj^{\prime }}\right)\rangle }_{q(B)}\right]=\exp\left[\Psi \left(w_{jj^{\prime }}^{B}\right)-\Psi \left(\sum_{j^{\prime }}w_{jj^{\prime }}^{B}\right)\right] \end{array}$$

(34) $$\begin{array}{cc} c_{ij}^{⋆} & ≜\exp\left[{\langle \ln\left(c_{ij}\right)\rangle }_{q(C)}\right]=\exp\left[\Psi \left(w_{ij}^{C}\right)-\Psi \left(\sum_{j}w_{ij}^{C}\right)\right] \end{array}$$

Ψ and 〈.〉 are the Digamma function and expectation, respectively. Moreover, we update the shape and scaled parameters as follows [100]:

(35) $$\begin{array}{c} {\bar{\alpha }}_{ijd}=\frac{u_{ijd}}{v_{ijd}} \end{array}$$

(36) $$\begin{array}{c} \langle \ln\left(\alpha_{ijd}\right)\rangle =\Psi \left(u_{ijd}\right)-\ln\left(v_{ijd}\right) \end{array}$$

(37) $$\begin{array}{c} {\bar{\beta }}_{ijd}=\frac{h_{ijd}}{\sum_{d=1}^{D}h_{ijd}} \end{array}$$

Here, we summarize the whole procedure of the SD-based HMM in Algorithm 1:  
**Algorithm 1:** Variational learning of the SD-HMM model.1.    Initialize the shape and scale parameters of the SD distribution.2.    Define the initial responsibilities.3.    Compute $w^{B},w^{C}$, and $w^{\pi }$.4.    Initialize B,C, and π.5.    while |old likelihood - new likelihood| ≥ 06.      Compute the data likelihood.7.      Compute the responsibilities with the forward–backward procedure.8.      Update the hyperparameters of the shape and scale parameters.9.      Update $w^{B},w^{C}$, and $w^{\pi }$ using responsibilities $\gamma^{B},\gamma^{C}$, and $\gamma^{\pi }$.10.    Update B,C, and π using $w^{B},w^{C}$, and $w^{\pi }$.

## 4. Experimental Results

In this part, we evaluate our model on publicly available datasets. Here, we explain the procedure and dataset: We propose a novel clustering algorithm and compare our model with other methods. Before applying our model to the datasets, we removed all labels and we did not have any training or testing. After predicting labels by our proposed clustering algorithm, we compared the actual and predicted labels with each other. To assess the performance, we applied four following metrics where TP,TN,FP, and FN represent the total number of true positives, true negatives, false positives, and false negatives, respectively:

(38) $$\begin{array}{cc} & Accuracy=\frac{TP+TN}{\mathrm{Total}\;\mathrm{number}\;\mathrm{of}\;\mathrm{observations}}\\ & Precision=\frac{TP}{TP+FP},\;Recall=\frac{TP}{TP+FN}\\ & F1-score=\frac{2\times precision\times recall}{precision+recall} \end{array}$$

Normalization of features: Considering various ranges of features, we applied min–max scaling with the following formula, which helps in shifting and re-scaling the values and keep them between zero and one:

(39) $$\begin{array}{c} \vec{X}=\frac{\vec{X}-{\vec{X}}_{min}}{{\vec{X}}_{max}-{\vec{X}}_{min}} \end{array}$$

We tested our model on publicly available and real datasets, the so-called opportunity [101,102] and UCI HAR databases [103].

### 4.1. Opportunity Dataset

In this dataset, data were collected by external and wearable sensors. Volunteers attached wearable sensors to their clothes and the rest were attached to objects or installed at points of interest. Through this setting, various activities of different levels were recognized. The sensors used were (1) body-worn sensors, including 7 inertial measurement units (IMUs), 12 3D acceleration sensors, and 4 3D coordinates from a localization system. (2) Object sensors, including 12 objects, were instrumented with wireless sensors measuring 3D acceleration and the 2D rate of turn. (3) Ambient sensors, including 13 switches and 8 3D acceleration sensors in kitchen appliances and furniture. Data were collected from 4 individuals and 6 runs per user with 5 activities of daily living and 1 “drill” run, which is a scripted sequence of activities. We analyzed four daily activities of individuals standing, walking, lying, and sitting. We tested our model on 2 runs of individual activities.

- Oversampling: Both datasets have 108 features; as illustrated in Table 1, there are considerable inequalities in the distribution of instances per cluster. As shown in the first run of the test, the percentages of four activities are 59.7%, 17.4%, 19.9%, and 3% for standing, walking, lying, and sitting, respectively. These shares for the second run are 41%, 23.8%, 5.1%, and 30%. As this challenge results in frequency bias, the model may be affected by the dominant class and learn from clusters, including more observations. We tackled this challenge with oversampling using a method called the synthetic minority over-sampling technique (SMOTE). In this method, new data points were generated by interpolating between observations in the original dataset. Thus, we obtain a balanced dataset. After this step, we had equal observations in 4 clusters with 22,380 and 10,379 in the first and second runs, respectively.
- Missing values: In both datasets, we have several missing values, which are shown in Table 2 for the first and second runs, respectively. This is a typical issue, especially when we work on real datasets. We replaced missing values with the median of each feature to minimize the effects of the outliers. As we mentioned, we have 108 features.

After tackling the above-mentioned challenges, we tested our algorithm on these two datasets. Here, SD-HMM, D-HMM, and GMM-HMM stand for scaled Dirichlet-based HMM, Dirichlet-based HMM, and Gaussian-based HMM, respectively.

### 4.2. UCI Dataset

To collect data, the activities of 30 individuals who were between 19 and 48 years were analyzed. Each volunteer performed activities while wearing a smartphone on the waist. With the help of an embedded gyroscope and accelerometer, information related to the 3-axial 3-axial angular velocity and linear acceleration were captured. To label the data, a camera was applied for manual annotation. Similar to previous experiments, we analyzed the standing, walking, lying, and sitting of individuals. This dataset includes 561 features.

## 5. Results and Discussion

In the experimental part, we validated the performance of our proposed algorithm on three real-world datasets and compared it with D-HMM, which is similar to our proposed model. Moreover, we compared it with GMM-HMM as the most widely used alternative. In the opportunity dataset, we focused on two subsets and the results are demonstrated in Table 3 and Table 4. As shown in Table 3, SD-HMM outperforms GMM-HMM by 89.33%, 86.54%, 85.51%, and 86.02% in accuracy, precision, recall, and F1-score, respectively. In Table 4, we present the outcomes of our test on the second dataset. Similar to the previous case, SD-HMM robustness is enhanced by 87.12%, 87.28%, 85.44%, and 86.35% in accuracy, precision, recall, and F1-score, respectively. In the next part of our experiment which is dedicated to the UCI dataset, Table 5 indicates that SD-HMM has behavior similar to two previous cases and provides better results with 86.17%, 85.05%, 86.83%, and 85.93% in accuracy, precision, recall, and F1-score, respectively. Scaled Dirichlet distribution has one more parameter compared to Dirichlet distribution; this character provides more flexibility to SD-HMM. The main finding from the comparison of our novel model against the conventional model is that our proposed model can be considered an alternative.

## 6. Conclusions

In this work, we proposed scaled Dirichlet-based hidden Markov models. Our principal motivation was the various natures of data and the fact that the assumption of Gaussianity could not be generalized to all cases and scenarios. In recent years, other alternatives have been evaluated and they may provide better flexibility in fitting data. Scaled Dirichlet distribution has two parameters that modify the shape and scale of distribution and this character assists us in modeling asymmetric and various skewed forms. Here, we assumed that the scaled Dirichlet distribution is the source of emission probability distribution. With such modifications and improvements in the structure of GMM-HMM, we may achieve some robustness. After constructing our model, we learned that variational inference provides results with reasonable computational time and accuracy. To validate our model, we tested our proposed methodology on three datasets of human activity recognition. As this application has been applied in numerous medical domains, we intended to demonstrate the robustness of our novel method in such a demanding domain. Datasets used in this test were collected by wearable, object, and environmental sensors. The results of our evaluation indicate that our proposed method outperforms D-HMM and GMM-HMM. In future work, we will study the activities of several individuals and test other alternatives as emission probabilities. Moreover, we believe our work is more robust to outliers and we will introduce feature selection in the future.

## Figures and Tables

**Table 1:** Activities in the first and second runs.

	Standing	Walking	Lying	Sitting
First run	60%	20%	17%	3%
Second run	41%	24%	5%	30%

**Table 2:** Number of missing values in the first dataset.

Feature	Numbers of Nan in Each Feature
1, 2, 3	454
4, 5, 6, 10, 11, 12, 28, 29, 30	20
13, 14, 15	92
19, 20, 21	1681
22, 23, 24	311
34, 35, 36	37,507

**Table 3:** Model performance evaluation results for the first dataset.

Method	Accuracy	Precision	Recall	F1-Score
SD-HMM	89.33	86.54	85.51	86.02
D-HMM	85.96	86.28	85.18	85.72
GMM-HMM	86.08	83.54	82.37	82.95

**Table 4:** Model performance evaluation results for the second dataset.

Method	Accuracy	Precision	Recall	F1-Score
SD-HMM	87.12	87.28	85.44	86.35
D-HMM	85.96	86.28	85.18	85.72
GMM-HMM	85.14	82.24	83.75	82.99

**Table 5:** Model performance evaluation results for the UCI HAR dataset.

Method	Accuracy	Precision	Recall	F1-Score
SD-HMM	86.17	85.05	86.83	85.93
D-HMM	85.22	84.38	85.57	84.97
GMM-HMM	84.31	82.47	82.33	82.39

## Data Availability

Datasets are publicly available and can be found in following links: https://archive.ics.uci.edu/ml/datasets/opportunity+activity+recognition, https://archive.ics.uci.edu/ml/datasets/human+activity+recognition+using+smartphones.
